# Integrated transcriptomic and metabolomic profiling reveals a JAZ-transcription factor network governing MeJA-induced flavonoid accumulation in *Scutellaria barbata*

**DOI:** 10.3389/fpls.2026.1928253

**Published:** 2026-08-28

**Authors:** Xiaokang Han, Xiangmin Deng, Ziwen Hu, Yuchun Wang, Yingying Fan, Zhijun Su, Xiuyuan Man, Min Niu, Shiqing Dong

**Affiliations:** 1 School of Traditional Chinese Medicine, Jiangsu College of Nursing, Huai’an, Jiangsu, China; 2 Jiangsu Key Laboratory for Eco-Agriculture Biotechnology Around Hongze Lake, Huai’an Key Laboratory of Biophysics Breeding, School of Life Science, Huaiyin Normal University, Huai’an, Jiangsu, China

**Keywords:** flavonoid biosynthesis, metabolomics, methyl jasmonate, *Scutellaria barbata*, transcriptomics

## Abstract

**Background:**

Methyl jasmonate (MeJA) is a potent elicitor of secondary metabolism in plants, but its regulatory role in flavonoid biosynthesis in *Scutellaria barbata* remains unclear.

**Methods:**

Here, we performed an integrated transcriptomic and metabolomic analysis of *S. barbata* seedlings following exogenous MeJA treatment, combining differential expression and correlation network approaches to identify candidate genes, metabolites, and transcription factors (TFs) associated with flavonoid accumulation.

**Results:**

MeJA treatment significantly promoted the accumulation of multiple flavonoids and upregulated key biosynthetic genes, including *Sbar2C282T9_CHS2, Sbar5A47T51_CHI*, and *Sbar9A209T130_C4H*. A total of 2,453 differentially expressed genes (DEGs) were identified, which were significantly enriched in flavonoid biosynthesis and plant hormone signal transduction pathways. Among these, 180 differentially expressed TFs were detected, with the ERF, MYB, bHLH, NAC, and bZIP families being markedly enriched. Integrative correlation analysis revealed 40 TFs whose expression patterns were strongly correlated with 19 flavonoids. TF binding site prediction further identified seven candidate TFs predicted to target flavonoid structural genes, including *Sbar2C282T9_CHS2* and *Sbar9A209T130_C4H*. Protein‑protein interaction prediction suggested that several of these TFs, particularly bHLH members, may interact with JAZ repressors and form intra‑family interaction clusters.

**Discussion:**

Collectively, these findings suggest a potential JA-responsive transcriptional regulatory network and point to a conserved JAZ-TF module that may modulate flavonoid biosynthesis in *S. barbata*. This study provides valuable insights into the regulatory mechanisms of secondary metabolism in medicinal plants and offers candidate gene resources for metabolic engineering and molecular breeding of high‑flavonoid varieties.

## 1 Introduction

*Scutellaria barbata* D. Don, known as “Ban-zhi-lian” in traditional Chinese medicine) is a perennial herb of the Lamiaceae family. It is primarily distributed in southern China and serves as a key component in numerous traditional Chinese medicinal formulations (Shim et al., 2016). The dried whole plant is used medicinally for the treatment of sore throat, severe pain, edema, jaundice, and snake bites. Its medicinal efficacy is attributed to its properties of clearing heat and toxins, activating blood circulation to dissipate blood stasis, and inducing diuresis to reduce edema. Modern pharmacological studies have further confirmed that *S. barbata* possesses multiple bioactivities, including antitumor, antioxidant, antibacterial, anti-inflammatory, antiviral, and immunomodulatory effects (Chen et al., 2020). Currently, research and applications related to its antitumor activity have mainly focused on crude extracts or extracted fractions of this herb (Dai et al., 2013b). For example, the total flavonoids from *S. barbata* have been shown to inhibit the growth of various tumor cells through several signaling pathways (Zheng et al., 2018). Scutellarein, a flavonoid monomer isolated from *S. barbata*, can selectively trigger mitochondrial-mediated intrinsic apoptosis in malignant cells (Shi et al., 2019). Moreover, related flavonoid constituents such as wogonin exhibit dual anticancer and anti-inflammatory activities (Ren et al., 2026); scutellarin inhibits the proliferation of breast cancer stem cells (Ma et al., 2024a); and baicalin ameliorates insulin resistance and regulates hepatic glucose metabolism (Xu et al., 2018). Collectively, these findings lay a solid foundation for further elucidating the pharmacodynamic material basis and mechanisms of action of *S. barbata*.

Flavonoids serve as the fundamental pharmacodynamic substances in *S. barbata* and have been established as critical markers for its quality evaluation (Cheng et al., 2024; Dai et al., 2013a).To date, more than 50 structurally distinct flavonoids have been identified from this herb, including baicalein, scutellarin, wogonin, naringenin, apigenin, eriodictyol, and luteolin (Chen et al., 2020). Analogous to its congeneric medicinal species *S. baicalensis*, flavonoid biosynthesis in *S. barbata* originates from phenylalanine and likely proceeds via two primary metabolic branches. The first is the classic pathway, which is sequentially catalyzed by key enzymes such as phenylalanine ammonia-lyase (PAL), cinnamate 4-hydroxylase (C4H), 4-coumaroyl-CoA ligase (4CL), chalcone synthase (CHS), and chalcone isomerase (CHI).This route ultimately produces 4′-hydroxyflavones, with scutellarein as the representative compound. The alternative pathway may involve the coordinated activity of specific cinnamate CoA ligase (CLL) and pinocembrin chalcone synthase (CHS-2).It directly employs cinnamic acid as a substrate to drive the biosynthesis of 4′-deoxyflavones with a chrysin skeleton, such as baicalein and wogonin. These flavonoid aglycones can be further modified via glycosyltransferases to form corresponding glycosidic derivatives, such as scutellarin and baicalin (Fang et al., 2023; Xu et al., 2020). These findings indicate that *S. barbata* harbors an intact metabolic network capable of synthesizing structurally diverse flavonoids. Accordingly, systematic clarification of its flavonoid biosynthetic pathways and regulatory mechanisms holds significant scientific importance for revealing the formation patterns of its pharmacodynamic substances, as well as for guiding quality improvement and sustainable utilization of this resource. The recent publication of the *S. barbata* genome (Li et al., 2023; Qiu et al., 2023) has laid a critical foundation for in-depth dissection of the molecular regulatory of flavonoid biosynthesis.

Jasmonates (JAs) are an important class of plant hormones that play a key role in regulating the synthesis of primary and secondary metabolites in plants (Du et al., 2025; Liu et al., 2023; Lv et al., 2024; Ma et al., 2018). Among them, MeJA has been widely validated as an effective elicitor that significantly promotes the accumulation of key secondary metabolites in various medicinal plants. In the absence of JA, JAZ proteins bind and inhibit TFs such as MYC2, silencing the expression of downstream target gene. Upon MeJA treatment, the bioactive conjugate JA-Ile is perceived by the COI1-JAZ co-receptor complex. This complex recruits the SCFCOI1 ubiquitin ligase to degrade JAZ proteins, thereby releasing TFs to activate transcriptional reprogramming of defense-associated genes (Zander and Vesper 2026; Lv et al., 2024; Xu et al., 2026). Although MeJA exhibits significant effects on regulating flavonoid biosynthesis across plant species, its regulatory mechanism governing the flavonoid biosynthetic pathway in *S. barbata* remains unclear.

Although recent advances in synthetic biology have enabled the *in vivo* production of baicalein and scutellarein in engineered *E. coli* and yeast (Xu et al., 2020), the identification and optimization of biological parts remain a critical bottleneck for the metabolic engineering of these flavonoids. In this study, we performed transcriptomic and metabolomic profiling of *S. barbata* under MeJA treatment to identify key metabolic and regulatory genes involved in flavonoid biosynthesis. We found that MeJA significantly induced the accumulation of multiple flavonoids, including hesperetin, dihydromyricetin, glycitin, luteolin, and apigenin, and upregulated the expression of biosynthetic pathway genes such as *Sbar3A3T168_CYP98A*, *Sbar5A47T51_CHI*, and *Sbar2C282T9_CHS2*. Integrative multi-omics analysis further revealed several TFs, including Sbar13C163T17 (MYB), Sbar4A177T104 (bHLH), Sbar6A24T143 (NAC), and Sbar9P227T23 (bHLH), that participate in the regulation of flavonoid biosynthesis. Moreover, protein interaction prediction suggested that these TFs may interact with SbarJAZs to play a key role in JA-mediated regulation of flavonoid accumulation. This study provides insights into the potential molecular mechanism of JA-regulated flavonoid biosynthesis in *S. barbata* and offers new perspectives on the transcriptional regulatory networks underlying secondary metabolism in medicinal plants.

## 2 Materials and methods

### 2.1 Plants and chemical treatments

Seeds of *S. barbata* were surface-sterilized and sown in sterilized nutrient soil (peat:vermiculite = 3:1, v/v). Seedlings were grown in a controlled growth chamber with a constant 22 °C, 16 h light/8 h dark photoperiod.

For chemical treatment, 30-day-old seedlings of uniform size were selected. MeJA was dissolved in a small volume of ethanol and then diluted with distilled water to a final concentration of 1 mM. The seedlings were treated by drenching the roots with the 1 mM MeJA solution, while control plants (CK) received an equal volume of distilled water. Aboveground tissues were harvested at 24 h post-treatment, with six biological replicates per group. All samples were immediately frozen in liquid nitrogen and stored at -80 °C for subsequent analyses. Transcriptome sequencing and metabolomic profiling were performed by Shanghai Majorbio Bio-Pharm Technology Co., Ltd.

### 2.2 Untargeted metabolomics analysis

Plant samples (100 mg) were frozen and ground, then mixed with 800 μL of extraction solvent (methanol:water = 4:1, v/v). The mixture was ultrasonically extracted at low temperature. After centrifugation, the supernatant was transferred to an autosampler vial with an insert for metabolomics analysis. Metabolite detection was performed using an ultra-high performance liquid chromatography coupled with quadrupole/electrostatic field orbitrap mass spectrometry system (UHPLC-Q Exactive, Thermo Fisher Scientific, USA) for LC-MS/MS analysis (Dong et al., 2025). For chromatographic separation, 3 μL of sample was injected onto a BEH C18 column (100 mm × 2.1 mm i.d., 1.7 μm, Waters Corporation, USA). Mobile phase A consisted of 2% acetonitrile in water containing 0.1% formic acid, and mobile phase B was pure acetonitrile containing 0.1% formic acid. The flow rate was 0.40 mL/min, and the column temperature was maintained at 40 °C. Mass spectrometry was performed using positive and negative ion scanning modes over a mass range of m/z 70-1050. The ion spray voltage was set to 3.5 kV in positive mode and -3 kV in negative mode. The sheath gas pressure was 50 psi, auxiliary gas pressure 13 psi, and ion source heating temperature 450 °C. A cyclic collision energy ramp of 20-40–60 V was applied. The MS1 resolution was set to 70,000, and the MS2 resolution to 17,500. During the analysis, samples were kept at 4 °C in the autosampler. To minimize instrumental fluctuations, samples were analyzed in a random injection order, and quality control (QC) samples were inserted periodically to monitor system stability and data reliability.

Raw data were processed using Progenesis QI software (Waters Corporation, USA), and a data matrix was generated by matching against an in-house plant metabolite database (MJDBPM). Data preprocessing was performed on the cloud.majorbio.com platform as follows: (1) variables with >20% missing values within a group were filtered out; (2) QC samples were normalized using the total peak area sum to eliminate systematic errors arising from sample processing, concentration differences, and instrumental bias; (3) variables with relative standard deviation (RSD) >30% were removed, and those with RSD ≤30% were retained for differential quantification; (4) log_10_ transformation was applied to correct heteroscedasticity, reduce data asymmetry, and improve normality. Principal component analysis (PCA) was conducted using the R package ropls (version 1.6.2). Differential metabolites were identified by combining univariate analysis (t-test, volcano plot) with multivariate statistical analysis (OPLS-DA/PLS-DA) and fold change (FC). The default thresholds were *P* < 0.05 and VIP > 1, with no mandatory filtering on FC (both FC < 1 and FC > 1 were accepted). Metabolite functional annotation was performed using the KEGG database (https://www.kegg.jp/kegg/compound/). Pathway enrichment analysis was carried out using the Benjamini–Hochberg (BH) method for multiple testing correction of *P*-values.

### 2.3 Relative expression analysis by qRT-PCR

Total RNA was extracted from 100 mg of *S. barbata* tissue powder using the RNAprep Pure Plant Kit (TIANGEN, China) according to the manufacturer’s instructions. First-strand cDNA was synthesized from 500 ng of total RNA using the Evo M-MLV Reverse Transcription Premix Kit (Accurate Biotechnology, Hunan, China). qRT-PCR was performed using SYBR Green Pro Taq HS (Accurate Biology, Hunan, China) and gene-specific primers (**Supplementary Table 1**). The relative transcript levels of target genes were calculated using the 2^-ΔΔCT^ method.

### 2.4 Transcriptome analysis

Total RNA was extracted from *S. barbata* tissues using the TRIzol method. RNA concentration, purity, integrity, and RQN value were assessed using a Nanodrop 2000, agarose gel electrophoresis, and an Agilent 5300, respectively. Library construction required a minimum of 1 μg total RNA with a concentration ≥30 ng/μL, RQN >6.5, and OD260/280 ratio between 1.8 and 2.2. mRNA was enriched using Oligo(dT)-conjugated magnetic beads and fragmented into approximately 300 bp fragments. cDNA was synthesized by reverse transcription, followed by end repair, A-tailing, adapter ligation, PCR amplification, and purification to obtain the final library. Paired-end sequencing was performed on the NovaSeq X Plus platform. Raw data were evaluated for base composition, quality distribution, and error rates. Adapter sequences, low-quality bases (Q<20) at the 3’ end, reads with N content >10%, and trimmed reads shorter than 20 bp were removed using fastp to generate high-quality clean reads. The clean reads were aligned to the *S. barbata* reference genome (version GWHAOTP00000000) (Qiu et al., 2023) using HiSat2 (Kim et al., 2015). Alignment quality was further assessed in terms of sequencing saturation, gene coverage, and read distribution across genomic regions. Gene expression levels were quantified using RSEM. Differentially expressed genes (DEGs) were identified using DESeq2 (Love et al., 2014) with the criteria of FDR <0.05 and |log2FC| ≥1. Functional annotation was performed using the GO and KEGG databases. Enrichment analysis was carried out using the Python scipy package, and multiple testing correction was performed using the BH (FDR) method, with a significance threshold of adjusted *P* < 0.05.

### 2.5 Integration analysis of metabolome and transcriptome

To elucidate the molecular mechanism underlying MeJA-induced flavonoid biosynthesis in *S. barbata*, differentially accumulated metabolites (DAMs) and differentially expressed TFs were integrated for correlation analysis. For each treatment group (MeJA and CK), three biological replicates were used for both transcriptomic and metabolomic analyses, and the data were paired by replicate (i.e., MeJA_1, MeJA_2, MeJA_3, CK_1, CK_2, CK_3). Within each paired replicate, the relative abundance of each DAM and the expression level of its corresponding TF were used to calculate the Pearson correlation coefficient to quantitatively assess their association. The correlation coefficients between metabolites and differentially expressed TFs were visualized using network diagrams.

## 3 Results

### 3.1 Exogenous MeJA treatment promotes flavonoid accumulation in *S. barbata*

**Figure 1:**
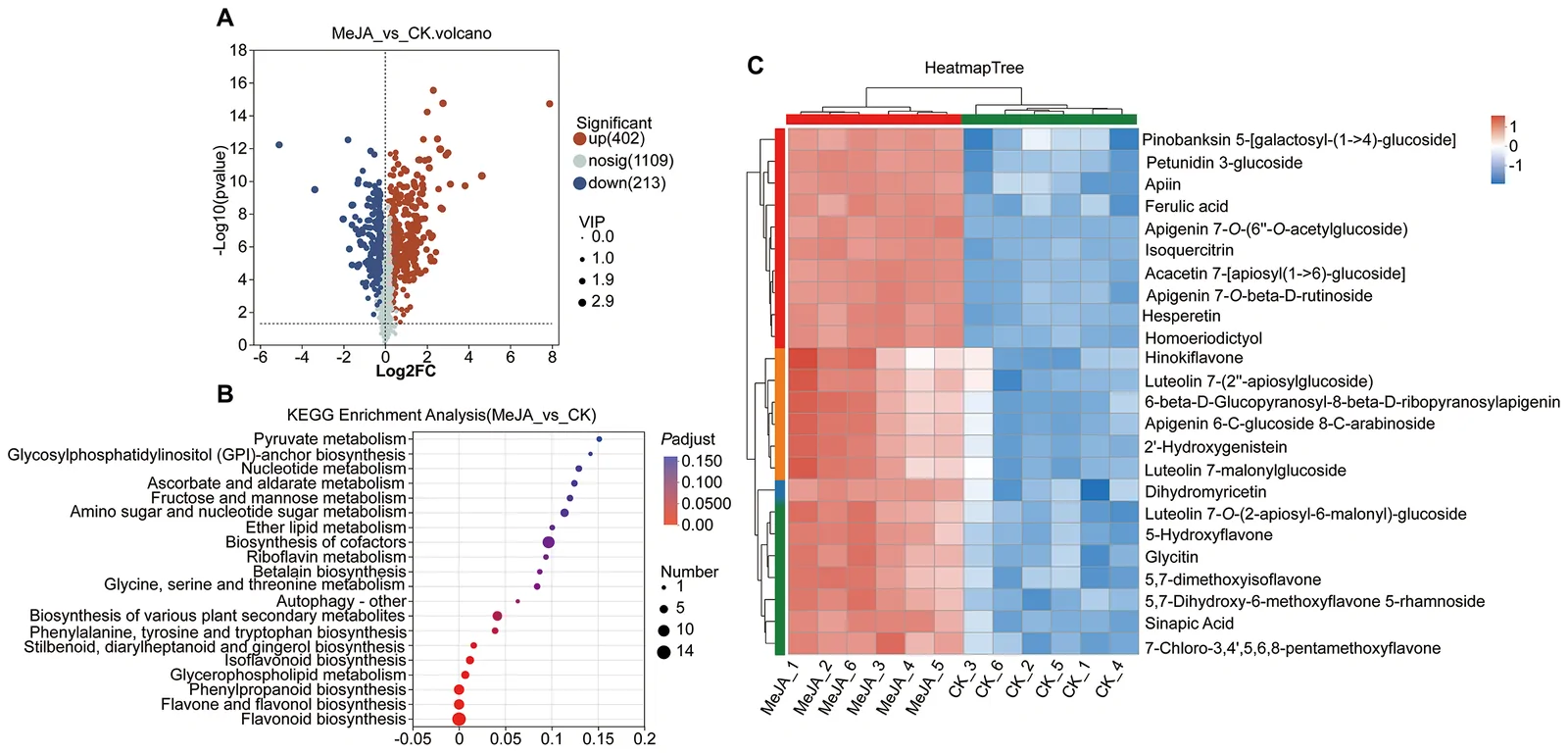
Analysis of DAMs in *S. barbata* under MeJA treatment. **(A)** Volcano plot of DAMs. Red dots represent significantly upregulated metabolites, blue dots represent significantly downregulated metabolites, and gray dots indicate non-significant metabolites. **(B)** Bubble plot of KEGG pathway enrichment. Bubble size represents the number of differential accumulated metabolites enriched in each pathway; the x-axis indicates the rich factor. **(C)** Heatmap of upregulated flavonoids. Red indicates upregulation, and blue indicates downregulation.

To investigate the effect of MeJA on secondary metabolite accumulation in *S. barbata*, an untargeted metabolomic analysis was performed on the MeJA-treated group (MeJA) and the control group (CK). Principal component analysis (PCA) revealed a distinct separation of metabolic profiles between the two groups (**Supplementary Figure 1**), with favorable model fitting parameters, indicating high reproducibility of the observed metabolic differences. A total of 1,724 known metabolites were identified under both positive and negative ion modes (**Supplementary Table 2**). Differential metabolite analysis showed that MeJA treatment significantly upregulated 402 metabolites and downregulated 213 metabolites (**Figure 1A**). KEGG pathway enrichment analysis further demonstrated that the differentially accumulated metabolites (DAMs) were significantly enriched in the flavonoid biosynthesis pathway (**Figure 1B**). Notably, the contents of various flavonoids, including hesperetin, homoeriodictyol, 2′-hydroxygenistein, dihydromyricetin, glycitin, 5-hydroxyflavone, luteolin, and apigenin, were markedly increased (**Figure 1C**; **Supplementary Table 3**). Collectively, these findings confirm that exogenous MeJA treatment significantly promotes the accumulation of bioactive flavonoids in *S. barbata*.

### 3.2 Transcriptomic analysis reveals MeJA-induced DEGs in *S. barbata*

**Figure 2:**
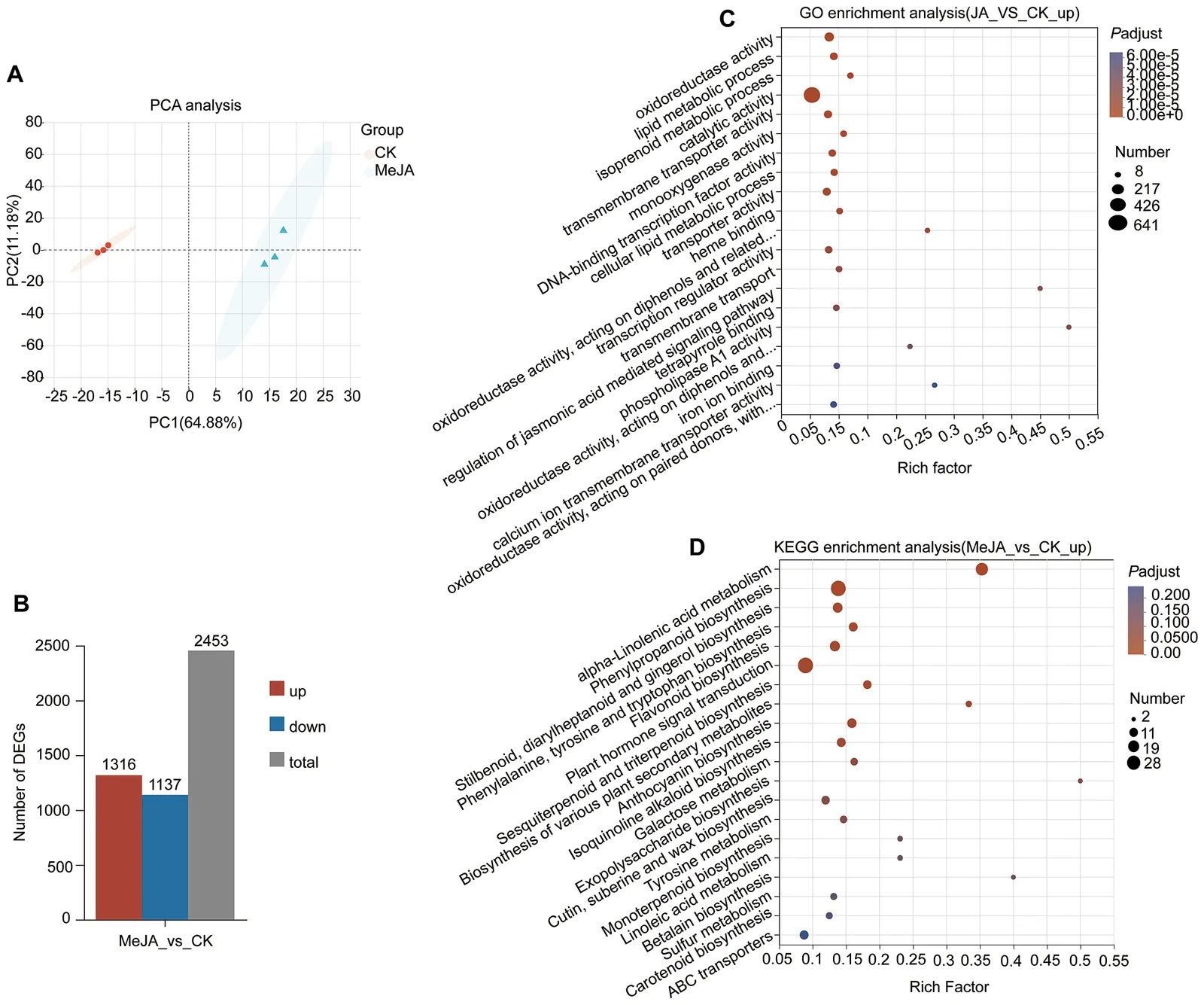
Analysis of DEGs in *S. barbata* under MeJA treatment. **(A)** PCA plot. The x-axis represents principal component 1 (PC1) and the y-axis principal component 2 (PC2); the percentages in parentheses indicate the proportion of variance explained by each principal component. **(B)** Bar chart of DEGs. Red bars represent upregulated genes, and blue bars represent downregulated genes. **(C)** Bubble plot of GO enrichment for upregulated genes (top 20 GO terms). The y-axis shows GO terms, and the x-axis shows the rich factor. Dot color corresponds to different ranges of adjusted *P*-values (*P*adjust < 1). **(D)** Bubble plot of KEGG pathway enrichment for upregulated genes (top 20 pathways). The y-axis shows pathway names, and the x-axis shows the rich factor. Dot size represents the number of genes annotated in the pathway, and dot color indicates different ranges of adjusted *P*-values (*P*adjust < 1).

To elucidate the molecular mechanism underlying MeJA-regulated flavonoid biosynthesis in *S. barbata*, RNA sequencing (RNA-seq) analysis was performed on seedlings from the MeJA-treated group (MeJA) and the control group (CK). A total of 39.27 GB of high-quality sequencing data were obtained. Using the *S. barbata* reference genome as a reference, the sequence alignment rate ranged from 97.65% to 97.82%. A total of 25,915 expressed genes were identified, including 23,392 annotated genes and 2,523 unannotated genes, which corresponded to 42,019 transcripts (21,132 annotated and 20,887 unannotated transcripts) (**Supplementary Table 4**). PCA showed that the first principal component (PC1), accounting for 93% of the total variance, clearly distinguished the MeJA and CK groups (**Figure 2A**). Biological replicates clustered closely within each group, indicating that MeJA treatment systematically remodels the transcriptomic landscape of *S. barbata*.

Differential expression analysis identified a total of 2,453 DEGs, including 1,316 upregulated and 1,137 downregulated genes (**Figure 2B**; **Supplementary Table 5**). GO functional enrichment analysis revealed that the upregulated DEGs were primarily enriched in terms related to catalytic activity, oxidoreductase activity, and DNA-binding transcription factor activity (**Figure 2C**; **Supplementary Table 6**). KEGG pathway enrichment analysis further demonstrated that these DEGs were significantly enriched in pathways such as flavonoid biosynthesis, plant hormone signal transduction, as well as sesquiterpenoid and triterpenoid biosynthesis (**Figure 2D**). Notably, genes involved in JA biosynthesis and signal transduction, such as *allene oxide cyclase* (*AOC*), *jasmonate-induced oxygenase* (*JOX*), and *jasmonate-ZIM domain proteins* (*JAZ*), were markedly upregulated upon MeJA treatment (**Supplementary Figure 2**).

### 3.3 MeJA treatment modulates expression of flavonoid biosynthetic genes

**Figure 3:**
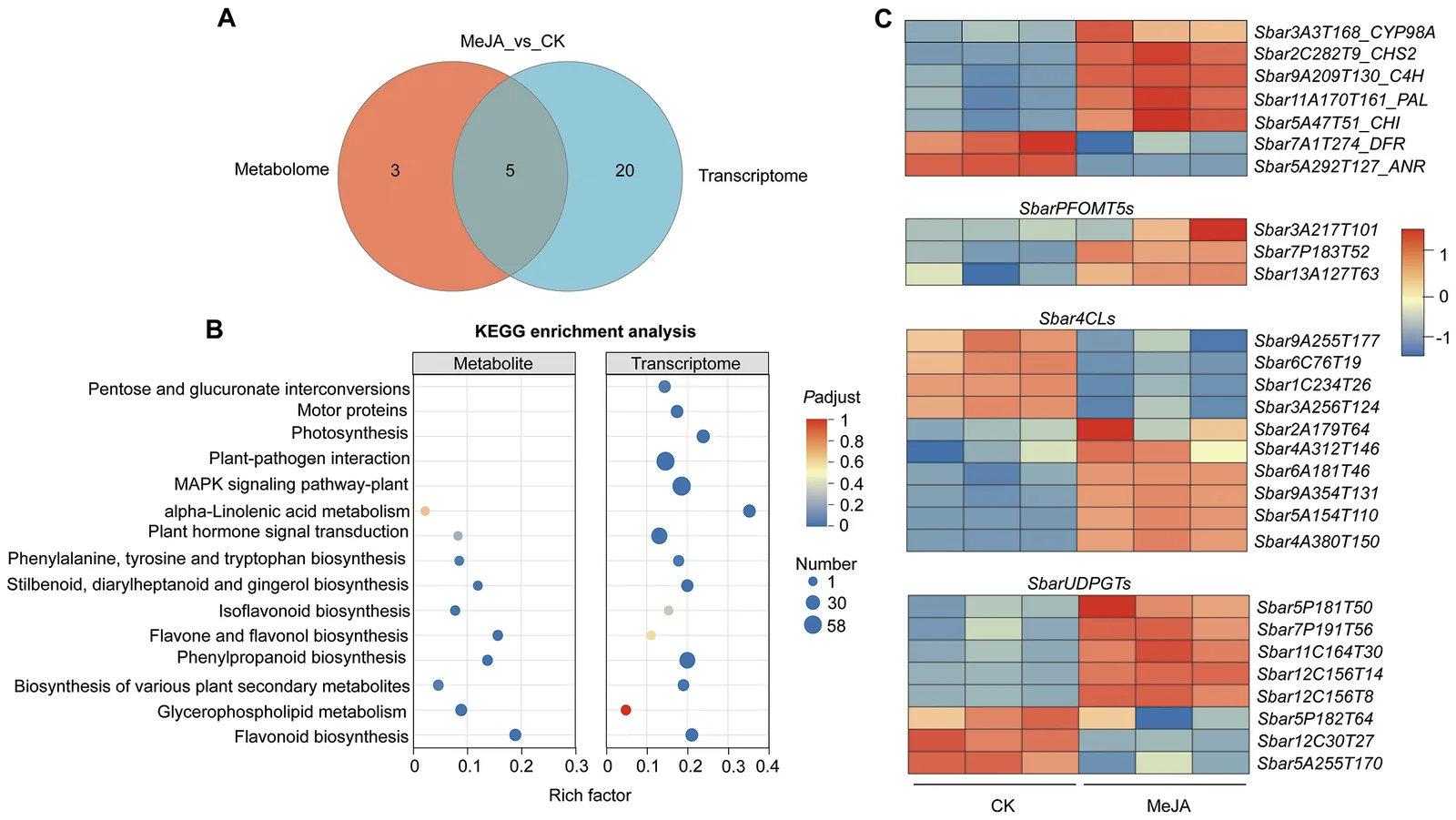
Integrative transcriptomic and metabolomic analysis of genes involved in flavonoid biosynthesis. **(A)** Venn diagram of shared pathways. Different colors represent different omics layers, and the overlapping area indicates pathways commonly enriched in both datasets. **(B)** Association bubble plot of KEGG pathways. The left side shows metabolomic results, and the right side shows transcriptomic results. Each bubble represents a pathway, and a larger bubble indicates a greater number of enriched genes or metabolites in that pathway. **(C)** Heatmap of differentially expressed genes in the flavonoid biosynthesis pathway. Red indicates higher gene expression levels in the sample, while blue indicates lower expression levels.

To investigate the transcriptional basis underlying MeJA-regulated flavonoid accumulation in *S. barbata*, an integrated transcriptomic and metabolomic analysis was performed. KEGG joint enrichment analysis of DEGs and DAMs in the MeJA_vs_CK comparison identified five significantly enriched pathways (**Figure 3A**): phenylalanine, tyrosine and tryptophan biosynthesis (map00400); stilbenoid, diarylheptanoid and gingerol biosynthesis (map00945); phenylpropanoid biosynthesis (map00940); flavonoid biosynthesis (map00941); and biosynthesis of other plant secondary metabolites (map00999) (**Figure 3B**).

Within the flavonoid biosynthesis pathway, key enzyme-encoding genes exhibited distinct expression patterns. Specifically, *Sbar11A170T161_PAL*, *Sbar2C282T9_CHS2*, *Sbar5A47T51_CHI*, and *Sbar9A209T130_C4H* were upregulated, whereas *dihydroflavonol 4-reductase* (*Sbar7A1T274_DFR*) and *anthocyanidin reductase* (*Sbar5A292T127_ANR*) were downregulated, a pattern that may reflect feedback regulation or a reallocation of metabolic flux toward downstream modification steps. Three homologs of *8-O-methyltransferase* (*PFOMT5*) showed increased transcript levels, while ten homologs of *4-coumarate:CoA ligase* (*4CL*) displayed a complex regulatory pattern, with some members upregulated and others downregulated (**Figure 3C**; **Supplementary Table 5**). Among the eight differentially expressed *UDP-glucosyl transferase* (*UDPGT*), five were upregulated and three were downregulated. To validate the reliability of the RNA-seq data, selected DEGs were subjected to qRT-PCR analysis. The qRT-PCR results were consistent with the transcriptomic trends, confirming high data accuracy and reproducibility (**Supplementary Figure 3**). These findings provide direct transcriptomic evidence supporting the role of MeJA in flavonoid accumulation.

### 3.4 Key transcription factors involved in MeJA-induced flavonoid biosynthesis

**Figure 4:**
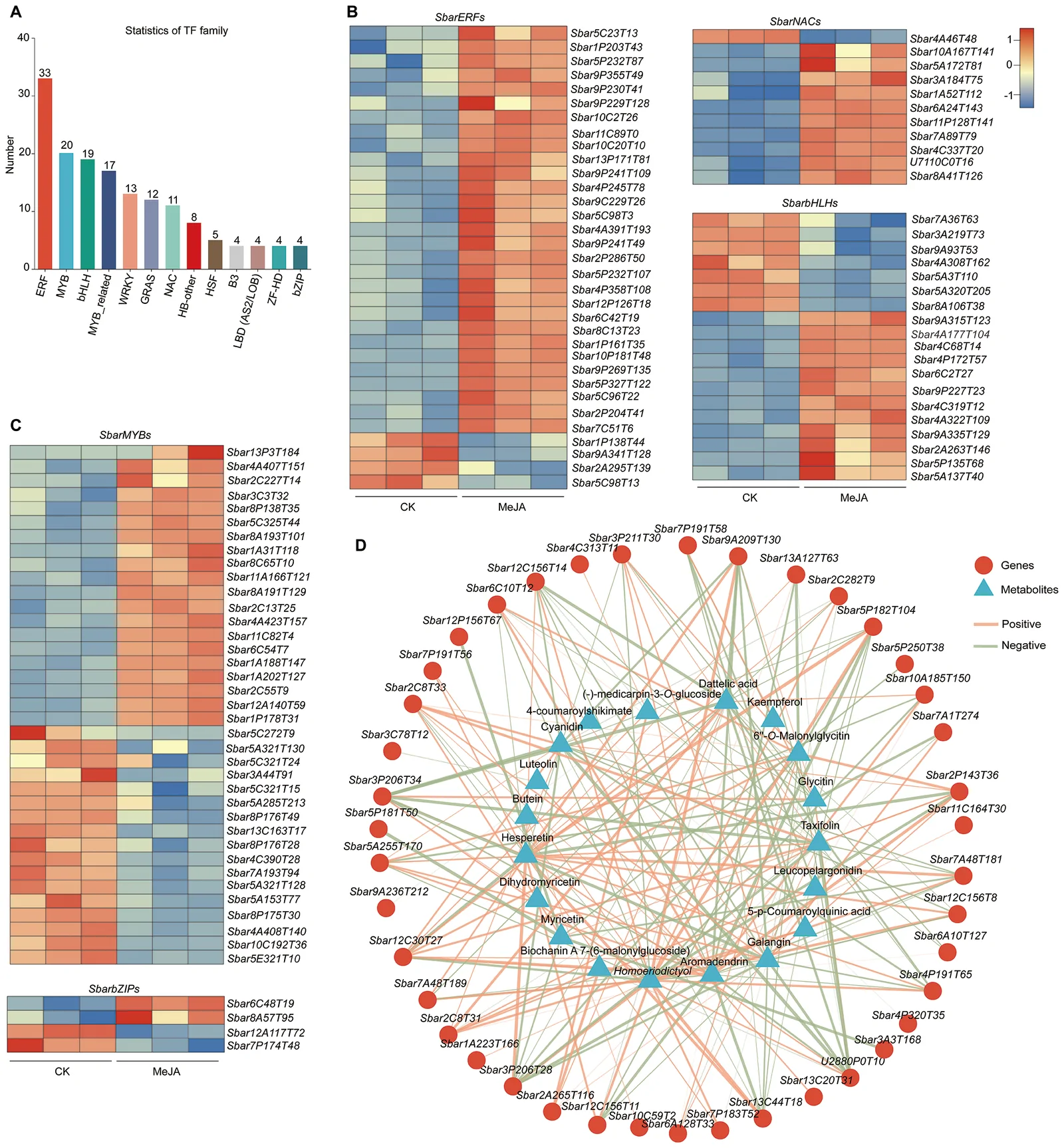
TFs involved in MeJA-induced flavonoid biosynthesis. **(A)**. Statistics of differentially expressed TF families. The x-axis represents different TF families, and the y-axis indicates the number of DEGs in each family. **(B, C)**. Heatmaps showing the expression profiles of DEGs from the ERF, NAC, bHLH, MYB, and bZIP families. Red indicates high expression, and blue indicates low expression. **(D)**. TF-metabolite co-expression network. Triangle nodes represent flavonoid metabolites, and circular nodes represent TF genes. Edges indicate co-expression relationships between TFs and metabolites, with red edges representing positive correlations (cor > 0) and green edges representing negative correlations (cor < 0). Edge thickness is proportional to the absolute value of the correlation coefficient, meaning thicker lines correspond to stronger correlations.

The biosynthesis of flavonoids is not only regulated by structural genes but also coordinately modulated by various transcription factors (TFs), with transcriptional regulation playing a central role in gene expression networks. Therefore, elucidating how TFs mediate MeJA responses and regulate the biosynthesis of bioactive compounds in medicinal plants is of great significance for molecular breeding and quality improvement. Based on transcriptomic analysis of *S. barbata* under MeJA treatment, a total of 180 differentially expressed TFs were identified, among which the *ERF*, *MYB*, *bZIP*, *bHLH*, *WRKY*, and *NAC* families were significantly enriched (**Figure 4A**; **Supplementary Table 7**). Further analysis revealed that these differentially expressed TFs included 33 *SbarERFs* (4 downregulated, 29 upregulated), 11 *SbarNACs* (1 downregulated, 10 upregulated), 19 *SbarbHLHs* (7 downregulated, 12 upregulated), 37 *SbarMYBs* (including MYB_related; 17 downregulated, 20 upregulated), and 4 *SbarbZIPs* (2 downregulated, 2 upregulated) (**Figures 4B, C**; **Supplementary Table 5**).

To investigate the potential regulatory relationships between the aforementioned TFs and flavonoid metabolites, we performed correlation analysis between differentially expressed TFs and differentially accumulated flavonoids, and displayed the top 200 strongly correlated pairs ranked by the absolute value of the correlation coefficient. Combining differential expression analysis with TF-metabolite co-expression network analysis, we found that 14 *SbarMYBs*, 9 *SbarbHLHs*, 8 *SbarERFs*, 6 *SbarNACs*, and 3 *SbarbZIPs* that showed potential associations with 19 flavonoids, respectively (**Figure 4D**; **Supplementary Table 8**). Specifically, *Sbar13C163T17* (*MYB*) was positively correlated with dihydrokaempferol and negatively correlated with hesperetin and homoeriodictyol; *Sbar5P327T122* (*ERF*) was negatively correlated with myricetin; *Sbar6A24T143* (*NAC*) was positively correlated with homoeriodictyol; *Sbar6C48T19* (*bZIP*) was positively correlated with hesperetin and homoeriodictyol, and negatively correlated with dihydrokaempferol, 6’’-*O*-malonylglycitin, cyanidin, taxifolin, and galangin; *Sbar7P174T48* (*bZIP*) was negatively correlated with xanthohumol; *Sbar4A177T104* (*bHLH*) was positively correlated with hesperetin and homoeriodictyol, but negatively correlated with dihydrokaempferol, taxifolin, and galangin; *Sbar9P227T23* (*bHLH*) was negatively correlated with myricetin (**Figure 4D**; **Supplementary Table 8**). These findings indicate that these differentially expressed TFs, particularly members of the bHLH family, are potentially correlated with the accumulation of multiple flavonoids. This exploratory association analysis provides testable candidate TFs and hypotheses regarding their potential involvement in MeJA-induced flavonoid biosynthesis in *S. barbata* and provide valuable clues for further identification and functional characterization of key regulatory factors.

### 3.5 Exploratory prediction of key transcription factors potentially involved in flavonoid biosynthesis

**Figure 5:**
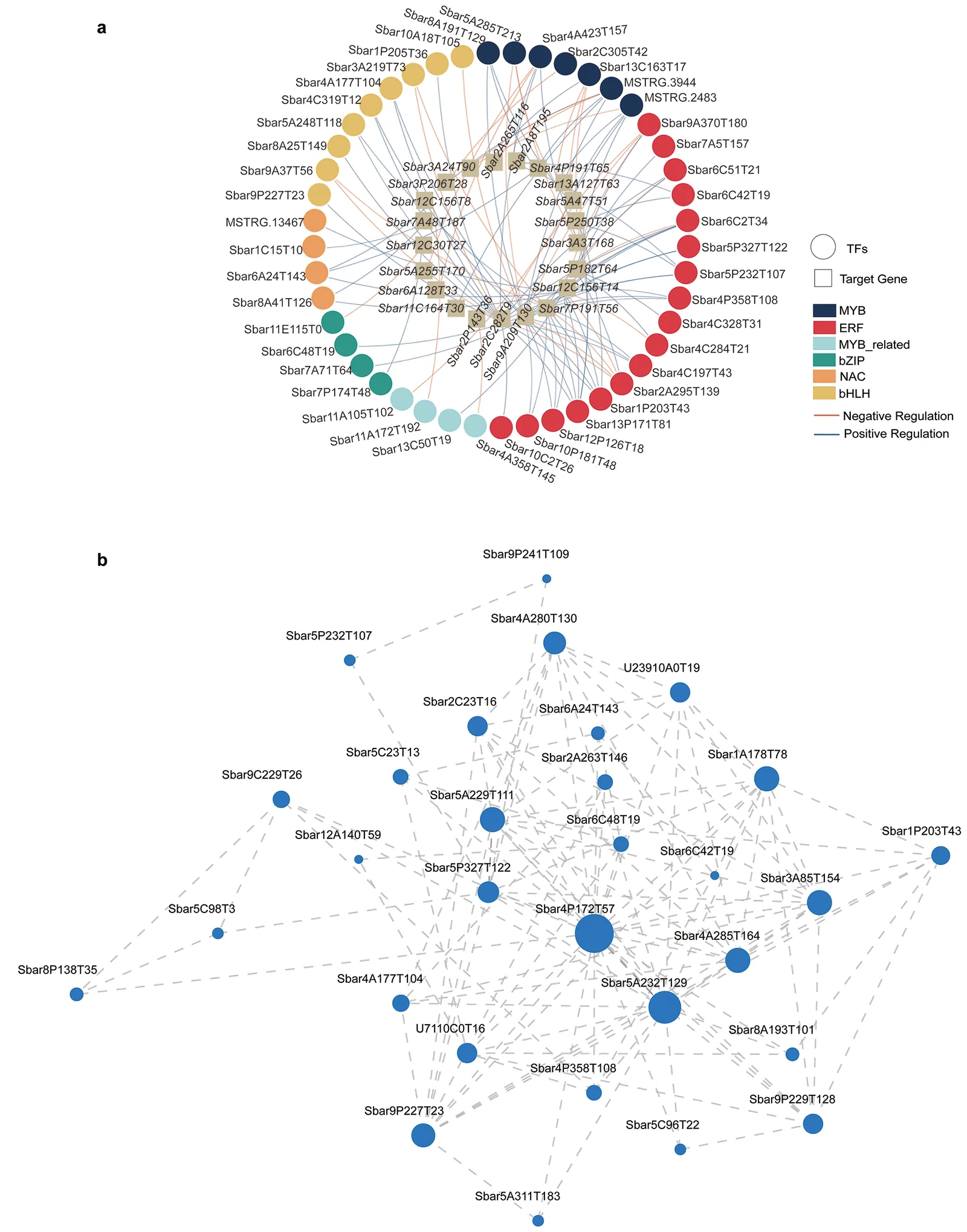
Regulatory network analysis of key TFs. **(A)** TF-target gene prediction network. Node shape and color: circular nodes represent TFs, and square nodes represent target genes. TFs are colored according to their family classification: ERF (red), MYB (dark blue), bHLH (golden yellow), NAC (orange), bZIP (cyan), and MYB_related (light blue). Edge colors indicate regulatory type: blue edges represent positive regulation (activation), and red edges represent negative regulation (inhibition). TF labels are placed outside the nodes, while target gene labels are placed insidesquare nodes. **(B)** Protein-protein interaction (PPI) network. Nodes represent proteins, and edges indicate interactions between them. Node size reflects the connectivity degree (the number of directly interacting partners) in the network; larger nodes correspond to higher degree, indicating greater importance of the protein within the network.

To explore potential transcriptional regulatory mechanisms underlying flavonoid biosynthesis in *S. barbata*, we performed a computational prediction of target genes for ERF, NAC, bHLH, MYB, and bZIP family TFs using FIMO (Grant et al., 2011) with a threshold of *P* < 0.05. The promoter regions analyzed spanned from 1000 bp upstream to 100 bp downstream of the transcription start site. This in silico prediction identified 171 significant TF-target gene regulatory pairs (**Figure 5A**; **Supplementary Table 9**), suggesting potential associations between these TFs and structural genes involved in the flavonoid pathway. Specifically, Sbar13C163T17 (MYB) was predicted to target four genes, positively regulating *Sbar13A127T63*_*PFOMT5* and negatively regulating *Sbar3A3T168*_*CYP98A*, and *Sbar5A47T51*_*CHI*. Sbar5P327T122 (ERF) was predicted to activate *Sbar9A209T130*_*C4H* and *Sbar7P191T56*_*UDPGT*. Sbar6A24T143 (NAC) positively regulate *Sbar12C156T14*_*UDPGT*, and *Sbar12C156T8_UDPGT*. Sbar6C48T19 (bZIP) upregulated *Sbar7P191T56*_*UDPGT*, while Sbar7P174T48 (bZIP) activated *Sbar12C30T27_UDPGT*. In addition, Sbar4A177T104 (bHLH) positively regulated *Sbar2C282T9_CHS2* and *Sbar9A209T130_C4H*, and Sbar9P227T23 (bHLH) positively regulated *Sbar2C282T9_CHS2* (**Figure 5A**; **Supplementary Table 8**, **S9**). These exploratory predictions provide candidate TFs and target gene pairs that may be involved in coordinating flavonoid accumulation in *S. barbata*, although experimental validation is required to confirm these regulatory interactions.

To further explore the potential links between JAZ proteins and the candidate TFs, we constructed a protein-protein interaction network using *Arabidopsis thaliana* as a reference. The network comprised 64 nodes and 140 edges (**Figure 5B**; **Supplementary Table 10**). Interaction analysis revealed that several candidate TFs are potentially connected to JAZ proteins or other JA pathway components. Sbar5P327T122 (ERF) was predicted to interact with multiple JAZ proteins (TIFY5A, TIFY10A, TIFY10B) and other ERF members (ERF017, ERF105, DREB1F). Sbar6A24T143 (NAC) showed potential connection to NAC002, which in turn connects to MYC2 and ABF2. Sbar6C48T19 (bZIP) directly interacted with ABF2. Sbar6C48T19 (bZIP) was predicted to associate with ABF2, which further connects to DREB2A, NAC002, MYC2, and MYB102. Sbar7P174T48 (bZIP) was only predicted to interact with bZIP42 (Sbar8A57T95). Sbar9P227T23 (bHLH) showed extensive predicted interactions with multiple JAZ proteins (TIFY5A, TIFY10A, TIFY10B, TIFY6B), as well as with bHLH13, bHLH92, and Sbar4A177T104 (bHLH), forming an intra-bHLH interaction cluster and suggesting a possible role as a node in JA signaling. Similarly, Sbar4A177T104 (bHLH) was predicted to interact with several JAZ proteins, as well as with bHLH13, bHLH92, and Sbar9P227T23 (bHLH). Notably, the predicted interaction between these two bHLH members hints at potential functional synergy downstream of the JAZ-MYC module (**Figure 5B**; **Supplementary Table 10**).

Overall, integrating in silico TF target prediction and protein-protein interaction analyses provides a set of testable hypotheses suggesting that the identified TFs may act downstream of JAZ repressors to potentially influence flavonoid biosynthetic genes, thereby possibly contributing to MeJA-induced flavonoid accumulation in *S. barbata*. Nevertheless, these predictions require experimental validation in future studies to confirm their functional relevance.

## 4 Discussion

### 4.1 MeJA induces flavonoid accumulation and biosynthetic gene expression in *S. barbata*

JA has been shown to promote flavonoid accumulation in diverse plant species. For example, MeJA induces *MdCHS*, *MdF3H*, and *MdUFGT* expression in apple, thereby enhancing anthocyanin biosynthesis (Sun et al., 2017). In *S. baicalensis*, MeJA treatment elevates4′-hydroxyflavones levels in roots and upregulates corresponding biosynthetic enzymes (Geng et al., 2023). Exogenous MeJA also markedly increases *ZbCHS* expression and flavonoid metabolite accumulation in *Zanthoxylum bungeanum* (Ma et al., 2024b). In the present study, exogenous MeJA significantly increased the contents of multiple flavonoids in *S. barbata*, including hesperetin, homoeriodictyol, 2′-hydroxygenistein, dihydromyricetin, glycitin, 5-hydroxyflavone, luteolin, and apigenin (**Figure 1**; **Supplementary Table 3**). Concurrently, several key flavonoid biosynthetic genes, such as *Sbar11A170T161_PAL*, *Sbar2C282T9_CHS2*, *Sbar5A47T51_CHI*, *Sbar9A209T130_C4H*, *Sbar13A127T63*_*PFOMT5*, and certain members of *Sbar4CL* family, were upregulated in response to MeJA (**Figure 3C**; **Supplementary Table 5**).

### 4.2 Transcriptional regulation of flavonoid biosynthesis is mediated by multiple TF families

TFs serve as master regulators of flavonoid biosynthesis by controlling structural gene expression, with MYB, bHLH, WRKY, NAC, and bZIP family members playing prominent roles (Zheng et al., 2026). MeJA promotes proanthocyanidin accumulation in *Dioscorea composita* via DcWRKY11 activation (Yu et al., 2025) and drives flavonoid glycoside production in *Ficus pandurata* through cytokinin-sucrose metabolic reprogramming (Yang et al., 2023). In *S. baicalensis*, SbMYB3 directly binds and activates the *SbFNSII-2* promoter to regulate baicalein biosynthesis (Fang et al., 2023). In *Citrus reticulata*, CrcMYBF1 transactivates *Crc1,6RhaT* to boost hesperidin accumulation (Su et al., 2025). In *Paeonia suffruticosa*, PsbHLH1 positively regulates anthocyanin biosynthesis by directly activating *PsDFR* and *PsANS* (Qi et al., 2020). Multi-omics analyses have further revealed synergistic modulation of flavonoid accumulation by bHLH2, bHLH8, and MYB5 in apricot fruit (Chen et al., 2023).

In the present study, TF members from multiple families, including ERF, NAC, bHLH, MYB, and bZIP, exhibited obvious expression changes in response to MeJA (**Figures 4B, C**; **Supplementary Table 5**). Integrative multi-omics analysis screened 40 TF genes (e.g., *SbarMYBs*, *SbarbHLHs*, *SbarERFs*, *SbarNACs*, and *SbarbZIPs*) whose transcript levels strongly correlated (positively or negatively) with diverse flavonoids, including aromadendrin, hesperetin, homoeriodictyol, taxifolin, cyanidin, and galangin (**Figure 4D**; **Supplementary Table 8**). Combined with TF binding site prediction, seven key regulators were further identified: Sbar13C163T17 (MYB), Sbar4A177T104 (bHLH), Sbar5P327T122 (ERF), Sbar6A24T143 (NAC), Sbar6C48T19 (bZIP), Sbar7P174T48 (bZIP), and Sbar9P227T23 (bHLH). These TFs were significantly associated with flavonoid biosynthetic genes such as *Sbar2C282T9_CHS2* and *Sbar9A209T130_C4H* (**Figure 5A**; **Supplementary Table 9**).

### 4.3 JAZ-TF interaction modules represent a conserved mechanism of JA-mediated flavonoid regulation

In the JA signaling cascade, JAZ proteins function as transcriptional repressors that interact with TFs to govern secondary metabolite biosynthesis (Liu et al., 2024; Xu et al., 2026). Several modules, including SmJAZ9-SmMYB76, SmJAZ9-SmbZIP5, SmJAZs-SmbHLH37/SmERF73-SmSAP4, and SmbHLH60-SmMYC2 have been shown to regulate tanshinone and salvianolic acid accumulation (Lv et al., 2024; Liu et al., 2025, 2023, 2022). Analogous JAZ-TF modules have been characterized in *Ginkgo biloba*, *Curcuma wenyujin*, and apple, where they integrate JA signals with light or gibberellin signals to modulate terpenoid or anthocyanin production (Zheng et al., 2024; Du et al., 2025; Wang et al., 2024; Ji et al., 2025). Collectively, these studies establish JAZ-TF interactions and TF-TF cross-talk as a conserved regulatory paradigm for JA-induced specialized metabolism.

In this study, protein-protein interaction prediction identified 64 interacting proteins and 140 interaction pairs. Several candidate TFs, including Sbar5P327T122 (ERF), Sbar9P227T23 (bHLH), and Sbar4A177T104 (bHLH), are potentially associated with JAZ proteins. Additionally, Sbar6A24T143 (NAC), Sbar6C48T19 (bZIP), and Sbar7P174T48 (bZIP) exhibit interactions with TFs from other or same family (**Figure 5B**; **Supplementary Table 10**). These findings provide initial insights into the complex modular mechanisms underlying JA-mediated flavonoid biosynthesis in *S. barbata*, establishing a foundation for future molecular dissection.

## 5 Conclusion

In this study, we demonstrated through integrated transcriptomics and metabolomics that MeJA systematically reprograms gene expression and metabolite profiles in *S. barbata*, leading to a marked enhancement of flavonoid accumulation. We identified a panel of differentially expressed TFs from the ERF, MYB, bHLH, NAC, and bZIP families; through correlation networks and target gene prediction, we pinpointed seven key regulators with strong associations to flavonoid structural genes. Notably, protein-protein interaction modeling revealed that several of these TFs, especially the bHLH pair Sbar9P227T23 and Sbar4A177T104, are likely positioned downstream of JAZ repressors, forming a core regulatory node that bridges JA signal perception with the transcriptional activation of flavonoid biosynthetic pathways. These findings establish a JAZ-TF-target gene framework in *S. barbata* that mirrors the conserved regulatory modules reported in *Salvia miltiorrhiza*, *Ginkgo biloba*, and other medicinal species, yet reveals unique features of the *S. barbata* flavonoid network. This work substantially advances our understanding of how JA signaling orchestrates specialized metabolism in medicinal plants and provides a valuable set of candidate genes and predicted interactions. Future functional validation of these regulatory modules will not only deepen the mechanistic dissection of flavonoid biosynthesis but also accelerate the rational development of *S. barbata* germplasm with enhanced levels of bioactive flavonoids for pharmaceutical applications.

## Data Availability

The raw RNA-seq reads generated in this study have been deposited in the NCBI Sequence Read Archive (SRA) and are accessible under BioProject accession number PRJNA1215018. The untargeted metabolomics raw data have been submitted to the Metabolomics Workbench repository and can be accessed directly via the project DOI: http://dx.doi.org/10.21228/M87K32.

## Supplementary material

Supplementary Figure 1 Principal Component Analysis (PCA)

Supplementary Figure 2 Expression of JA-related genes under MeJA treatment

Supplementary Figure 3 Validation of RNA-seq data by qRT-PCR

Supplementary Table 1 Primers used for the qRT-PCRs

Supplementary Table 2 Total ion count and identification statistics table

Supplementary Table 3 Differential metabolite statistics

Supplementary Table 4 Statistical table of sequencing data

Supplementary Table 5 Gene expression level

Supplementary Table 6 Statistics of GO enrichment analysis of differentially expressed genes

Supplementary Table 7 Statistics of differentially expressed TF families

Supplementary Table 8 Putative correlations between TFs and flavonoid metabolites

Supplementary Table 9 Predicted target genes of transcription factors involved in flavonoid biosynthesis in *S. barbata*

Supplementary Table 10 Protein-protein interaction network of JAZ proteins and candidate TFs in *S. barbata*
