# Supplementary material for: Integrated transcriptomic and metabolomic profiling reveals a JAZ-transcription factor network governing MeJA-induced flavonoid accumulation in Scutellaria barbata
Source: Front Plant Sci. 2026 Aug 28;17:1928253. doi: 10.3389/fpls.2026.1928253 (PMC13561975; doi:10.3389/fpls.2026.1928253)
Supplement: Supplementary file 1 [file DataSheet1.docx]

**Figure S1. Principal Component Analysis (PCA)**

**Figure S2. Expression of JA-related genes under MeJA treatment**

Note: The x-axis represents different comparison groups, and the y-axis represents the corresponding number of up-regulated and down-regulated genes. Red indicates up-regulation, and blue indicates down-regulation.

**Figure S3. Validation of RNA‑seq data by qRT‑PCR**

Relative expression levels of selected genes were measured by qRT‑PCR using *SbarActin* as the internal reference. RNA‑seq data (gray bars) and qRT‑PCR results (black bars) are shown for comparison. Values are presented as mean ± SD (*n* = 3 independent biological replicates). **P* < 0.05, ***P* < 0.01 indicate statistically significant differences between the MeJA‑treated and control groups (Student’s t‑test).
